# Posttranslational N-glycosylation of the hepatitis B virus large envelope protein

**DOI:** 10.1186/1743-422X-4-45

**Published:** 2007-05-30

**Authors:** Carsten Lambert, Reinhild Prange

**Affiliations:** 1 Institute for Medical Microbiology and Hygiene, University of Mainz, Augustusplatz, D-55101 Mainz, Germany

## Abstract

**Background:**

The addition of N-linked glycans to proteins is normally a cotranslational process that occurs during translocation of the nascent protein to the endoplasmic reticulum. Here, we report on an exception to this rule occurring on the hepatitis B virus (HBV) large L envelope protein that is a subject to co-plus posttranslational N-glycosylation.

**Results:**

By using an improved detection system, we identified so far unrecognized, novel isoforms of L. Based on mutational analyses, the use of N-glycosylation inhibitors, and pulse-chase studies, we showed that these isoforms are due to posttranslational N-glycan addition to the asparagines 4 and 112 within the preS domain of L. While an inhibition of N-glycosylation and glycan trimming profoundly blocked virus assembly and release, the posttranslational N-glycosylation of L itself was found to be dispensable for HBV morphogenesis.

**Conclusion:**

These data together with previous results implicate that the N-glycosylation requirements of virion release are due to functional inhibition of cell glycoproteins engaged by HBV.

## Background

N-glycosylation is known to possess important functions for many proteins, as it affects protein folding, quality control, sorting, degradation, secretion, and modulates immune responses [1,2]. N-linked glycans are added to proteins en bloc in the lumen of the endoplasmic reticulum (ER) as presynthesized oligosaccharides. The reaction is catalyzed by the oligosaccharyltransferase (OST)-complex that links the glycan unit to the asparagine residue in the target sequence N-X-T/S. As the OST-complex is associated with the translocon, N-glycosylation normally occurs cotranslationally when the nascent protein chain grows into the ER lumen [1,2]. However, in the present study we show that the large envelope protein of the hepatitis B virus (HBV) is also modified by posttranslational N-glycosylation.

**Figure 1:**
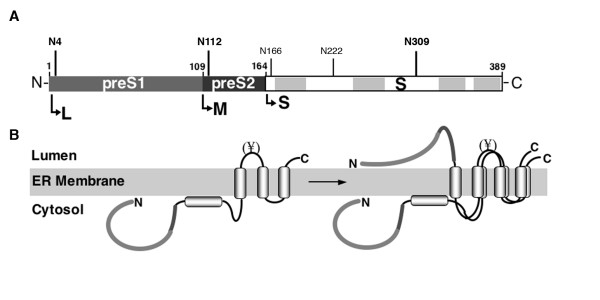
**Domain structure and transmembrane topology of the HBV L envelope protein**. (**A**) Schematic representation of L consisting of the preS1, preS2 and S domains. Numbers above the domains refer to the corresponding amino acids positions. The usage of the second and third start codons located at positions 109 and 164 of L leads to synthesis of the M and S proteins, respectively, as denoted by arrows. Consensus sequences for N-glycosylation are indicated by the corresponding asparagine (N) residue. (**B**) Mixed topology of L at the ER membrane. Upon cotranslational membrane integration, the preS1 and preS2 domains of L are initially located on the cytosolic surface of the ER (*Left*). During maturation, about ~50% of the L molecules posttranslationally translocate their preS region into the ER lumen (*Right*). Partial cotranslational N-glycosylation occurring at N309 is indicated by (¥).

HBV encodes three envelope proteins, the L (large), M (middle), and S (small) proteins, within one single open reading frame by using three different in-frame translation start codons and a common stop codon [3] (see Fig. 1A). Accordingly, the sequence of the S protein is repeated at the C-termini of M and L, which contain the additional N-terminal preS2 or preS2 plus preS1 domains, respectively. On biogenesis, all three proteins are cotranslationally integrated into the ER membrane by the topogenic signals of the S region concomitant with partial cotranslational N-glycosylation in their S domains [4,5]. The M protein is additionally modified by cotranslational N-glycosylation in its preS2 domain that specifically mediates its interaction with the ER lectin calnexin [6], likely for glycan-dependent quality control. In difference, thus far no N-glycan addition had been recognized to occur in the preS1 and preS2 (preS) domains of the L protein, although L also interacts with calnexin [7]. The absence of preS-linked N-glycosylation had been attributed to the unusual topogenesis of L, as its N-terminal preS domain is retained on the cytosolic side of the ER membrane during cotranslational translocation. Upon maturation, about half of the L molecules then posttranslationally translocate their preS region into the ER lumen thereby generating a dual transmembrane topology with bitopical essential functions, like envelopment of the viral nucleocapsid and receptor binding during HBV entry [8-11] (see Fig. 1B).

HBV formation strictly depends on a functional N-glycosylation apparatus of the cell, since both the inhibition of N-glycosylation and N-glycan processing by ER glucosidases block virion egress [12-14]. By contrast, the release of empty envelope subviral particles that are mainly formed by the S protein and known to accompany HBV morphogenesis does not require N-glycans [6,13]. Based on these observations, the preS2-specific N-glycan of the M protein was suggested to be the prime target for N-glycosylation inhibitors that would provoke aberrantly folded M polypeptides with the potential to block HBV envelope and hence virus maturation [14]. Consistent with this, a mutational inactivation of the preS2-specific N-glycosylation sequon of M has been shown to prevent virus release in one report [14]. Inconsistently, however, several studies have documented that M is dispensable for HBV morphogenesis [15-17] and thus leave the puzzle why virus production may require the preS2-linked glycan but does not need the M protein.

In this study we readdressed the role of N-glycosylation in HBV maturation with special emphasis on the L envelope protein. We provide first evidence that L is N-glycosylated within its preS1 and preS2 domains in an uncommon posttranslational manner. In order to solely examine the impact of the preS-specific glycosylation of L on virus production, we used an engineered *in vitro*-replication system that allows to study HBV assembly in the absence of an ongoing M protein synthesis. We found that mutational inactivation of the L-specific glycosylation sites did not affect virus maturation, whereas inhibitors of N-glycosylation and glycan trimming blocked virus production, thus indicating that proper N-glycosylation and processing of host cell components are involved in HBV multiplication.

## Results

### Detection of two novel L forms

**Figure 2:**
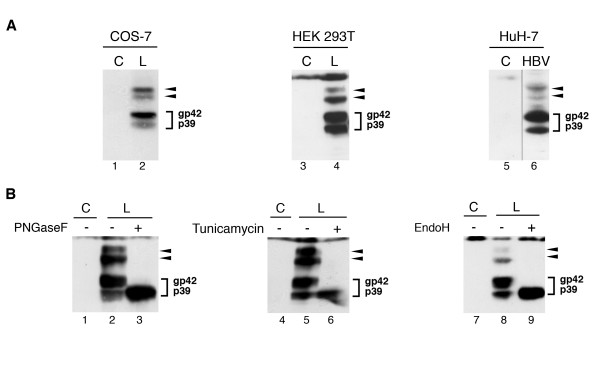
**L is synthesized in differently modified forms due to N-glycosylation**. (**A**) COS-7 cells and HEK 293T cells were either transfected with empty control plasmids (control, C) or transfected with vectors carrying the cloned L gene (L). HuH-7 cells were transfected with a cloned replication-competent HBV genome (HBV). Two days after transfection, microsomal vesicles were prepared and analyzed by SDS-PAGE and L-specific immunoblotting with the preS1-specific monoclonal antibody MA18/7. (**B**) The EcR-LHA-293 cell line with inducible L expression was either mock-treated (control, C) or induced with PonA. Cell lysates were mock-digested or digested with PNGase F, tunicamycin, or Endo H, as denoted above each panel. Samples were resolved by SDS-PAGE and subjected to L-specific immunoblotting. The nonglycosylated p39 and single-glycosylated gp42 forms of L are indicated on the right of each panel, and arrows to the right point at the two novel forms of L.

In previous works we have been focusing on the dual topology of the HBV L protein that is generated by partial posttranslational translocation of its N-terminal preS region across the ER membrane. In order to analyze the orientation of preS relative to the membrane, we repeatedly used protease protection assays of microsome-associated L chains [9,11,18]. The utilization of microsomal fractions purified from transfected cells instead of crude cell lysates enabled us to identify so far undetected forms of L. When microsomal fractions of COS-7 cells transiently expressing the wild-type L protein were analyzed by Western blotting with the L-specific antibody MA18/7, L appeared in its characteristic doublet of a 39-kDa nonglycosylated (p39) and a 42-kDa single-glycosylated (gp42) species due to partial cotranslational N-glycosylation in its S domain as expected (Fig. 2A). Importantly, we could detect two additional forms of L with molecular weights of about 48 kDa and 51 kDa that were missing in control-transfected cells. To rule out that the appearance of these novel forms was simply due to L synthesis in monkey cells, the L expression profile was analyzed in human HEK 293T cells transiently transfected with pNI2.L. Thereby, we could confirm the presence of the four specific L isoforms (Fig. 2A). To finally exclude that these forms resulted from an artificial overexpression of L in those cell lines, we investigated its expression pattern in human liver cells, the natural host cells of HBV. Therefore, HuH-7 cells were transfected with a cloned, replication-competent HBV genome that drives L synthesis under the control of its authentic promoter [19]. As shown in Fig. 2A, the four L-specific bands were again evident thus indicating that they present bona fide L isoforms.

### Novel L forms result from N-glycosylation

By using protease protection assays, we observed that the novel L forms were resistant against proteolysis and hence should have their preS domains orientated into the ER lumen (data not shown). Therefore, we reasoned that the higher molecular weights of the 48 kDa and 51 kDa L derivatives might be due to modification with N-glycans attached to the preS region that contains two potential consensus motifs (N4 and N112; Fig. 1A). To test for N-glycosylation, lysates of L-expressing HEK 293T cells were treated with Peptide:N-Glycosidase F (PNGase F), an enzyme that cleaves high mannose, hybrid and complex oligosaccharides from N-linked glycoproteins. As shown in Fig. 2B, PNGase F not only converted gp42 but also the two additional forms to the nonglycosylated p39 form of L. To verify these results, we analyzed the L expression profile in the presence of tunicamycin, which prevents N-glycosylation. As expected, treatment of L-expressing HEK 293T cells with tunicamycin inhibited the formation of gp42, but, importantly, also the generation of the 48 kDa and 51 kDa forms. Although these data pointed to differently N-glycosylated L chains, the 48 kDa and 51 kDa forms could equally represent a single-glycosylated L polypeptide carrying distinct oligosaccharide moieties due to processing of glycan structures in the Golgi stacks. To examine whether the L forms had acquired complex glycan chains, cellular extracts were treated with endoglycosidase H (Endo H) which cleaves N-glycans from the high-mannose-type present on glycoproteins in the ER, while glycans trimmed within the Golgi to complex/hybrid structures are resistant to Endo H. This analysis clearly revealed that all modified L forms were converted by Endo H to the non-glycosylated p39 L polypeptide (Fig. 2B), thus indicating that they did not resemble Golgi-processed glycan derivatives. Together, these data demonstrated additional N-glycosylation of L occurring beyond its known glycosylation site at N309.

### Novel L forms result from N-glycosylation within preS1 and preS2

**Figure 3:**
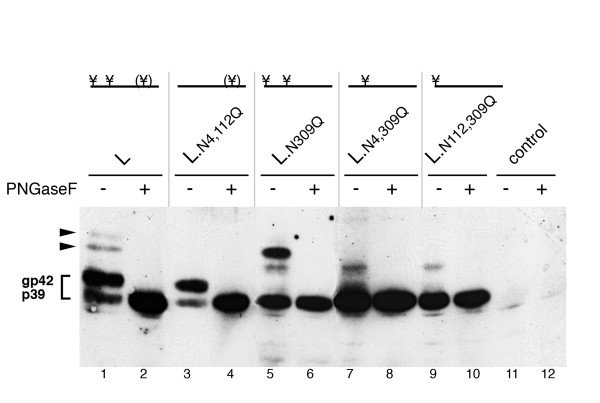
**N-glycosylation motifs within preS1 and preS2 of L are modified by N-glycosylation**. HEK 293T cells were either mock-transfected (control) or with the wild-type and mutant L constructs, as denoted above each panel. Diagrams (*Top*) show the N-glycosylation sites (¥) remaining for each mutant and brackets ((¥)) indicate known partial usage at N309. Following transfection, cell lysates were mock-treated or digested with PNGase F prior to SDS-PAGE and L-specific immunoblotting analysis.

We next were interested to identify the N-glycan attachment sites of L. Inspecting the primary sequence of L, we noted the existence of three consensus motifs within its S domain (N166, N222, N309) and two within preS (N4, N112) (see Fig. 1A). Because the asparagine corresponding to N309 of L is the only glycosylation target used in the S protein and in the S region of M [20], a modification at N166 or N222 of L appeared unlikely. Therefore, we focused on the two motifs within its preS1 and preS2 region of L (N4, N112) and replaced both putative asparagine acceptor residues by glutamines. After transient expression of the wild-type L and L.N4, 112Q double mutant in HEK 293T cells, lysates were mock-treated or treated with PNGase F prior to L-specific immunoblotting. As shown in Fig. 3, the combined mutation of the two preS-specific glycan acceptor sites of L resulted in the disappearance of the 48 kDa and 51 kDa forms. Unlike the wild-type, the L.N4, 112Q mutant was made in only non- and single-glycosylated forms (p39/gp42) with the latter likely being modified at N309, as shown by PNGase F treatment. To unequivocally determine whether both potential preS targets became modified, the positions N4 and N112 were mutated and analyzed individually. For facilitating the interpretation of the results, the S-specific glycosylation site of L (N309) was mutated in addition. The sole inactivation of this site (L.N309Q) yielded the non-glycosylated p39 and two glycosylated forms of about 45 kDa and 48 kDa, thus adding further evidence for preS-linked glycosylation of L. Next, the individual N4 or N112 mutations were combined with the N309Q substitution (L.N4, 309Q and L.N112, 309Q, respectively), thereby leaving only one preS-specific glycosylation site in each mutant. Both L.N4, 309Q and L.N112, 309Q mutants appeared in the non-glycosylated p39 and one glycosylated gp45 form, indicating that each of the two N-glycosylation sites within the preS domain of L became modified. Of note, however, the N-glycans attached to N4 or N112 have higher molecular weights than the glycan linked to N309.

### N-glycosylation within preS1 and preS2 occurs posttranslationally

**Figure 4:**
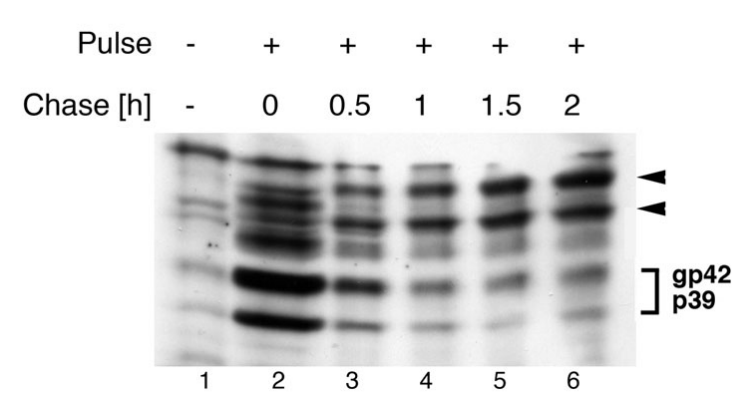
**N-glycosylation within the preS domain occurs posttranslationally**. The inducible EcR-LHA-293 cell line was either mock-treated (lane 1) or treated with PonA for 3 h to pulse-induce L synthesis. After PonA removal, L was chased for the indicated time periods in the presence of cycloheximide, and lysates were analyzed by SDS-PAGE and L-specific Western blotting. The nonglycosylated p39 and single-glycosylated gp42 forms as well as the newly discovered isoforms of L are indicated at the right.

Since translocation of the preS domain of L into the ER lumen proceeds in a posttranslational manner, we reasoned that preS-linked N-glycosylation could occur in a posttranslational mode. Addressing this point, we performed a kinetic analysis by using a recently established stable EcR-LHA-293 cell line with controllable L expression [21]. The expression of L was pulse induced for 3 h and the synthesized L population was then chased for different time periods in the presence of the protein synthesis inhibitor cycloheximide. As shown in Fig. 4, L synthesized during the pulse mainly appeared in its non-glycosylated p39 and single-glycosylated gp42 forms due to partial cotranslational N-glycosylation within its S domain. Conversely, during chase, the intensities of these bands decreased concomitant with the appearance of the glycosylated 48 kDa and 51 kDa forms modified within preS. Notably, the amount of these forms increased during chase extensions which demonstrated that N-glycans were added to N4 and N112 of L in an unconventional posttranslational reaction.

### Inhibition of N-glycosylation in the absence of the M protein inhibits HBV release

**Figure 5:**
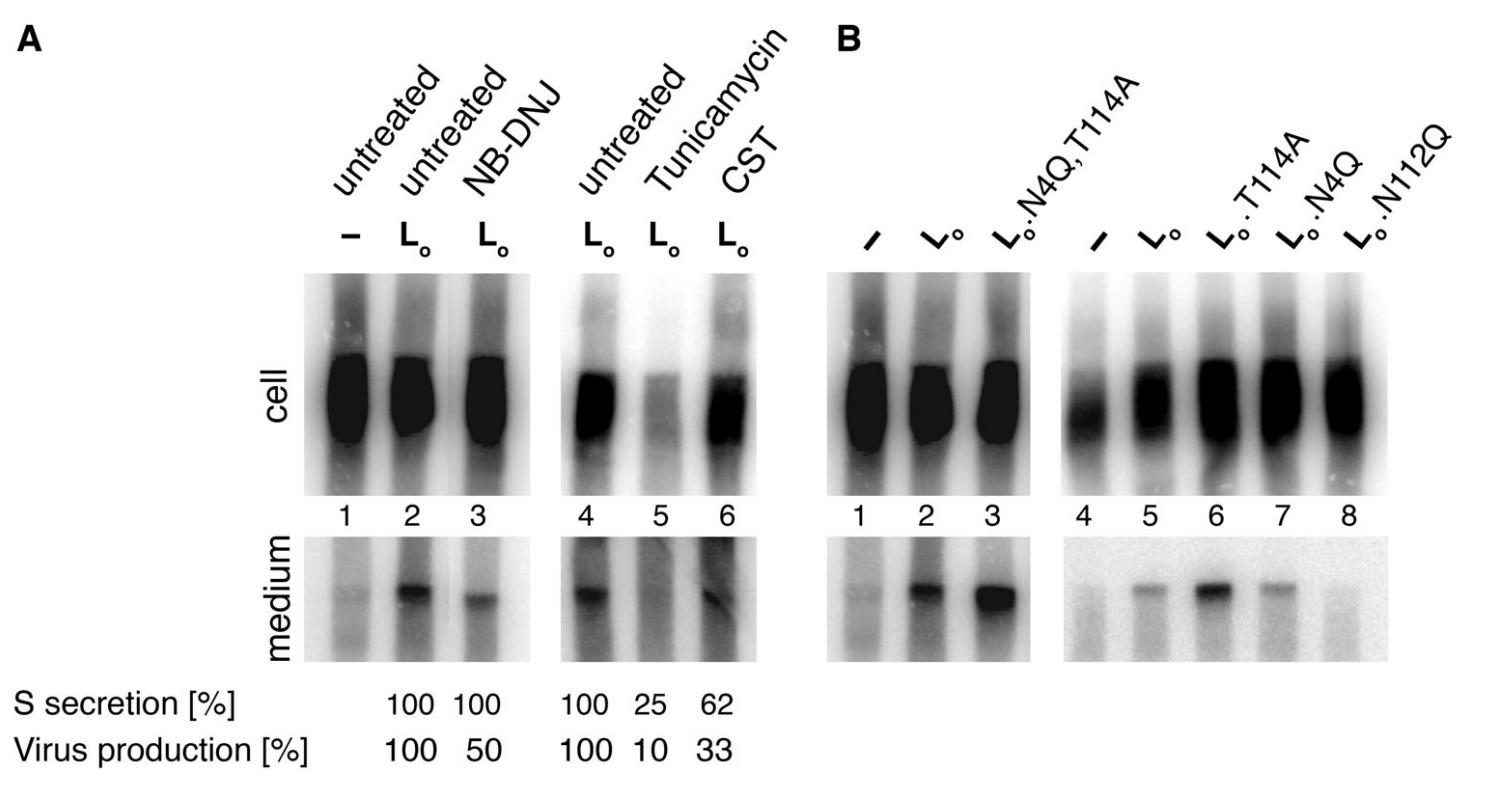
**Systemic but not preS-specific inhibition of N-glycosylation reduces virus production**. (**A**) To investigate the impacts of glycosylation inhibitors on HBV formation in the absence of the M protein, HuH-7 cells were (co)transfected with a plasmid-based HBV genome defective in envelope protein synthesis (pHBV.env^-^), a vector encoding the L gene with inactivated start codons for M and S (L_o_), plus an S expression vector. Upon transient expression, cells were treated with the indicated drugs for 70 h. Secretion of enveloped virions in the culture medium was detected by immunoprecipitation and radioactive labelling of the viral genome by the endogenous viral polymerase. The migration of the genome was visualized by agarose electrophoresis and PhosphorImaging (*Bottom*). Nonenveloped cytosolic nucleocapsids were immunoprecipitated from cell lysates and processed as above (*Top*). The amounts of virions released into the media were quantitated by measurement of the corresponding band intensities and demonstrated in % amount relative to corresponding mock-treated cells (Virus production). The amounts of subviral envelope particles harvested from the media were measured by an S-specific ELISA and depicted as above (S secretion). (**B**) Complementation of the env-negative HBV genome with L_o _mutants carrying single or double mutations in the preS-specific N-glycosylation sequons. HuH-7 cells were cotransfected with pHBV.env^-^, S, and the indicated wild-type or mutant L_o _constructs, and intracellular nucleocapsid assembly (Top) and virus release (Bottom) were analyzed essentially as in A.

A previous report had shown that HBV release can be blocked by mutation of the preS2-specific glycosylation site (N112) within the viral envelope open reading frame [14]. Because preS2-linked glycosylation of L was undiscovered thus far, the observed block to virus release had been attributed to an aberrant M protein defective in its preS2-specific glycan that might act in a dominant negative manner during HBV envelope formation. However, this proposal is in seemingly conflict with the observation that M is dispensable for HBV production [15-17]. With regard to the novel mechanism of posttranslational L glycosylation described herein, we rather asked if preS2-specific glycosylation of L is instead involved in HBV morphogenesis. To unequivocally address this point, it was important to study HBV production in the absence of an ongoing M expression. Therefore, we used transfection of a cloned replication-competent HBV genome that is deficient to drive envelope protein synthesis (HBV.env^-^). This genome was complemented in *trans *by cotransfection with S and L expression vectors. To prevent M synthesis from the complemented L expression vector, the start codon of M was likewise ablated in this construct (L_o_). With this approach, we analyzed the effects of inhibitors of the N-glycosylation pathway, like tunicamycin and the α-glucosidase inhibitors castanospermine (CST) or N-butyl-deoxynojirimycin (NB-DNJ), on HBV maturation. Virus formation and release from transfected HuH-7 cells were monitored by immunoprecipitation of cellular supernatants with envelope-specific antisera, radioactive labelling of the partially double-stranded viral genome by the endogenous polymerase of the virus, and detection of the isolated genome by agarose gel electrophoresis. Similarly, intracellular nucleocapsid assembly was monitored by using capsid-specific antibodies for immunoprecipitation of cellular lysates prior to endogenous polymerase reaction (EPR). The HBV.env^- ^construct *trans*-complemented with S alone was able to produce intracellular nucleocapsids but failed to support virion formation as expected (Fig. 5A). However, virus formation could be rescued by the coexpression of L_o_, thus confirming that M is dispensable for HBV production. Next, transfected cells were subjected to inhibitors of the N-glycosylation machinery. To control the fitness of the cells under drug treatment, the secretion of HBV subviral S particles was monitored by ELISA, as the production and export of such particles have been shown to remain unaffected by tunicamycin or α-glucosidase inhibitors [13]. As shown in Fig. 5A, prevention of N-glycosylation by tunicamycin almost completely blocked virus release but also the secretion of S as well as the production of intracellular nucleocapsids which hinted to cytotoxic effects of this drug. By contrast, the application of the α-glucosidase inhibitors NB-DNJ and CST had no or only modest cytotoxic effects under the assay conditions used, as evidenced by proper nucleocapsid assembly within such treated cells (Fig. 5A). Importantly, the level of virus export was strongly reduced by treatment with both NB-DNJ and CST inhibitors. Together, these results demonstrated that accurate N-glycosylation and glycan processing are essential for efficient HBV production even in the absence of the M envelope protein.

### Inhibition of posttranslational N-glycosylation of L does not inhibit HBV release

This prompted us to investigate whether posttranslational preS N-glycosylation of L per se is involved in HBV morphogenesis. As above, the HBV genomic construct defective in envelope protein synthesis (HBV.env^-^) was cotransfected together with wild-type or mutant S and L_o _expression constructs in HuH-7 cells and intracellular nucleocapsid assembly and release of virions into the medium were monitored by the EPR assay. Because the preS2-specific glycan acceptor residue (N112) of L is located within a critical region involved in nucleocapsid envelopment comprising amino acids 92 to 113 (corresponding to 103 to 124 in HBV, subtype adw2) [17,22], we created the substitution T114A that destroyed the glycosylation consensus motif (N-X-S/T) without being part of the nucleocapsid envelopment region. While the L_o_.N112Q variant had a negative phenotype as expected, the L_o_.T114A mutant still supported virus production and thus even more efficiently as compared to the wild-type L_o _protein (Fig. 5B). Similarly, inactivation of the preS1-specific glycosylation site of L had no inhibitory effect on virus formation, neither alone (L_o_.N4Q) nor in combination with the preS2 glycan knockout mutation (L_o_.N4Q, T114A) (Fig. 5B). In accordance with published data [14] we also found no influence on virion secretion when the glycan acceptor residue in the S protein (N146Q) and in the S domain of L (N309Q) was mutated simultaneously (data not shown). Together, these results indicated that posttranslational N-glycosylation of L as well as cotranslational N-glycosylation of L and S are not required for HBV maturation. Hence, the observed inhibitory effects of the N-glycosylation-interfering drugs on virus production are likely due to functional inhibition of host cell factors engaged by HBV for its propagation.

## Discussion

In this work we report that the HBV L envelope protein is modified by N-glycosylation within its N-terminal preS1 and preS2 domains. As would be expected from the unique L biogenesis and topology, these modifications take place after completion of L synthesis and thus in a posttranslational mode. In general, N-glycans are cotranslationally transferred to the asparagine residue of the sequon N-X-S/T as the growing polypeptide is translocated from the ribosome into the ER lumen [1,2]. However, recent reports provided first hints for an exception to this rule in such that N-glycans can be also added posttranslationally to polypeptides at least under artificial conditions. For examples, posttranslational N-glycosylation had been observed for the aglycoinsulin receptor synthesized in the presence of tunicamycin [23], for a truncated form of the peptidylglycine α-amidating monooxygenase [24], or in manosylphosphoryldolichol-deficient mutant cells [25]. The human coagulation factor VII is another substrate of posttranslational N-glycosylation which becomes modified within its very C-terminal region, presumably because conformational constraints do not favour a cotranslational N-glycan addition [26]. In the case of the HBV L protein, the posttranslationally N-glycosylated sites are located in its N-terminal domain but first entered the ER lumen after chain termination [9,11,18]. Hence, the data presented here pointed out the potential of the cellular machinery to conduct N-glycan attachment independently of protein translation. Although the enzymes catalyzing the posttranslational N-glycosylation of L remain to be characterized, it is tempting to speculate that the cellular OST-complex is responsible for this modification based on our mutagensis analysis, the use of endoglycosidases and the use of tunicamycin, an inhibitor of the OST-driven N-glycosylation reaction. Accordingly, completed L polypeptides that posttranslationally translocate their preS domains into the ER should still have access to the OST-complex even after their uncoupling from the ribosome. In support of this view, recent data suggested that the OST-complex might associate with the translocon in either a ribosome-dependent or ribosome-independent manner [27]. Therefore, it seems possible that proteins which remain transiently associated with the Sec61 translocon after chain termination or even reassociate with this channel can become N-glycosylated by OST. Based on pulse-chase analysis, we have evidence that the HBV L protein indeed is associated with the translocon for a prolonged time (our unpublished observations), thus rendering this protein as a model substrate to further investigate the mechanism(s) of posttranslational N-glycosylation uncoupled from protein synthesis.

Because the L protein along with its split topology is a key player in the HBV life cycle, and because N-glycans have been shown to serve as folding and trafficking devices of many glycoproteins [1,2], it appeared reasonable to assume that the newly identified posttranslational N-glycosylation of L may contribute to viral replication. Previous studies have well documented an extreme sensitivity of HBV egress to inhibition of N-glycosylation and ER glucosidase function. Mutational analyses of the S-specific N-glycan site, modified in all three envelope proteins, revealed that this site is dispensable for viral particle release and therefore should not be targeted by N-glycosylation-interfering drugs. Rather, the preS2-specific N-glycan of M was claimed to be responsible for N-glycan-dependent HBV maturation, as an inactivation of this sequon by a single mutation (N-X-T to N-X-A) blocked virus release [14]. Conversely, however, in two other reports a disruption of this sequon showed no measurable effects on virus production [17,22]. With regard to these conflicting data of the role of the preS2-specific N-glycan and of the M protein per se played during virus formation [15,16], here we have examined the impact of posttranslational N-glycosylation of L in HBV-replicating cells in which M protein synthesis was blocked. A treatment of these cells with the glucosidase inhibitors CST or NB-DNJ significantly reduced HBV release without, notably, affecting intracellular nucleocapsid assembly, implicating that these drugs interfered with proper L protein folding, trafficking and/or nucleocapsid envelopment. To our surprise, however, the mutational inactivation of the preS-specific N-glycosylation sites of L, either individually or in combination, did not diminish virus production. Together, these results suggested that posttranslational N-glycosylation of L itself is not necessary for HBV morphogenesis. Hence, the N-glycosylation requirements of virion release are likely due to modifications of cell glycoproteins assisting in L envelope and/or virus maturation. The search for such proteins is under active investigation.

Rather, the posttranslationally added N-glycans may serve other, yet to be determined functions in the HBV infection cycle. Interestingly, in an early study characterizing the L protein in secreted HBV particles, Heermann and co-workers also detected protein bands of > 45 kDa in purified virus samples but not in preparations of subviral particles [31]. Based on this observation it is tempting to speculate that L proteins with posttranslational glycan additions in the preS-domain may be selectively incorporated into mature virion particles. With respect to L topogenesis, these glycan chains presumably are exposed on the virus surface where they may contribute to virulence. First experiments analyzing the glycosylation pattern of secreted viral particles confirmed the existence of additional preS1-specific N-glycans (data not shown). For several viruses, like HIV-1, Sindbis virus, Dengue virus, human cytomegalovirus, hepatitis C virus, and Ebola virus, the N-linked glycans in their envelope glycoproteins have been shown to mediate host-cell recognition [28], and ref. therein]. Therefore, one putative function of the preS-linked glycans may reside in facilitating HBV binding to liver cells. In support of this view, a deletion of amino acids 3–7 of L, including the preS1 N-glycan sequon, has been shown to block HBV infection of primary human hepatocytes [10]. Synergistically, the preS-linked glycans may protect the surface-exposed preS domain of L along with its essential receptor binding site for degradation, thereby enhancing virus stability and supporting its infectivity. It is equally possible that these glycans may provide a means to combat humoral immune surveillance in such that they may form a formidable barrier for development of strong antibody responses to HBV.

## Methods

### DNA constructs

The mammalian expression vectors carrying the HBV (subtype ayw) L gene (pNI2.L) or S gene (pNI2.S) under the control of the human metallothionein IIA promoter have been described [11]. To abolish concomitant expression of the HBV M and S proteins from the L open reading frame, their translational start codons had been inactivated in the plasmid pNI2.L_o _as described [29].

Site-directed mutagenesis was performed using the QuickChange II XL kit (Stratagene) as recommended by the manufacturer. For simplification, amino acid numbering used herein will refer to consecutive numbering of the L protein rather than to the envelope protein domains (see Fig. 1A). The N112Q mutation was introduced with the forward primer 5'-CATCCTCAGGCC**C**TGCAGTGG**C**A**A**T CCACAACCTTC-3' (nucleotides (nts) 1210–1245, mutations are in boldface) and a complementary reverse primer. For the T114A mutation, the primer 5'-CCATGCAGTGGAA**C**TCC**G**CAACCTTCCACC-3' (nts 1220–1249) and its corresponding reverse primer were used. The N4Q mutation was introduced by PCR in a two-step reaction. First, a 150 bp product was generated using the 5'-primer 5'-CCTCCTCCAAGTCCCAGCGAACCC-3' (nts 772–795) and the mutagenic 3'-primer 5'-GCTGGTGGA**C**AG**C**T**G**CTGCCCCAT-3' (nts 895–921). In a second reaction, this product served as a 5'-primer together with the 3'-primer 5'-TGTGATGTTCTCCATATGCAGCGC-3' (nts 1378–1401). The resulting product was cut with *Sac*I and *Xho*I and used to substitute for the wild-type fragment of pNI2.L_o_. The mutation of N309Q within the S coding region (corresponding to N146Q of S) has been described [30]. A *Xho*I-*Spe*I-fragment containing the N309Q mutation (nts positions 1359–19011) was subcloned into the pNI2.L_o _vector.

### Cell culture, transfection, and lysis

In order to rule out cell type-specific effects on protein biogenesis, we used transient expression of L in different cell lines. COS-7 cells, HEK 293T cells, and HuH-7 liver cells were transfected using electroporation, Lipofectamine 2000 reagent (Invitrogen), or Lipofectamine/Plus reagents (Invitrogen), respectively, as recommended by the manufacturers. In addition, L synthesis and maturation was examined in the cell line EcR-LHA-293 [21] which stably expresses L under the control of the ecdysone-inducible promoter. Unless otherwise indicated, cell lysates were prepared by incubating the cells with lysis buffer (100 mM Tris-HCl (pH 8.0), 100 mM NaCl, 10 mM EDTA, 1% NP-40) for 15 min on ice and cleared by centrifugation at 10,000 *g *for 5 min at 4°C. Alternatively, cells were disrupted by homogenization and microsomal fractions were prepared by ultracentrifugation essentially as described [6].

### Glycosidase and tunicamycin treatment

For Peptide:N-Glycosidase F (PNGase F) treatment, cell lysates were boiled for 10 min in denaturing buffer (0,5% SDS, 40 mM DTT), and sodium phosphate (pH 7.5) and NP-40 were added to give a final concentration of 50 mM and 1%, respectively. Samples were divided in two portions and incubated for 1 h at 37°C with or without 1,000 U PNGase F (New England Biolabs). For endoglycosidase H (Endo H) digestion, cells were lysed in 50 mM Tris-HCl (pH 7.5), 150 mM NaCl, 0,5% SDS, 1% 2-mercaptoethanol and boiled for 10 min. Sodium acetate (pH 5.0) was added at a final concentration of 50 mM and split samples were incubated over night at 37°C with or without 5 mU Endo H (Roche). For the tunicamycin experiment, EcR-LHA-293 cells were treated with 10 μM of the inducer Ponasterone A (PonA, Sigma) for 15 h in the presence or absence of 5 μg/ml tunicamycin (Sigma).

### Pulse-chase-analysis

To pulse induce L protein synthesis, EcR-LHA-293 cells were incubated with 10 μM PonA for 3 h. After removal of the inducer, the cells were chase treated with 0,25 mM cycloheximide (ICN biochemicals) for different time periods. For preparation of microsomes, cells were washed twice with Tris-buffered saline (TBS, 50 mM Tris-HCl (pH 7.5), 150 mM NaCl) and incubated for 15 min on ice with 0,1 × TBS-buffer. Microsomes were prepared by homogenisation and ultracentrifugation as described [11]. The microsomal pellet was lysed in loading buffer and subjected to SDS-PAGE and immunoblotting with an L-specific monoclonal antibody recognizing an epitope within the preS1 domain of L (MA18/7) [31].

### Drug treatment of HBV-replicating cells

For replication of HBV in the HuH-7 liver cell line, plasmid HBV.env^- ^was used carrying an 1.1-mer of the HBV DNA genome [19] in which the translational start codons for the L, M, and S envelope protein had been inactivated by mutagenesis [29]. To allow virus production, pNI2-based plasmids encoding HBV envelope proteins were complemented *in trans*. For cotransfections, either plasmids pNI2.S plus pNI2.L_o _or pNI2.S plus glycosylation mutants of L_o _were employed. Cells were left untreated or treated for 70 h with 100 μg/ml N-butyl-deoxynojirimycin (NB-DNJ, Sigma), 1 mM castanospermine (CST, Applichem), or 5 μg/ml tunicamycin. Thereafter, cell culture supernatants were harvested and measured for the release of subviral/viral particles by ELISA (Auszyme, Abbott). In parallel, intracellular nucleocapsids and extracellular virions were isolated by capsid- or envelope-specific immunoprecipitations, respectively, prior to detection of the encapsidated viral progeny DNA by radioactive labelling of the partially double-stranded genome with 10 *μ*Ci [α^32^P]dATP (GE Healthcare) by the endogenous polymerase as described [29]. After extraction of the labelled DNA genomes from the immunoprecipitated samples, they were resolved on agarose gels and visualized by PhosphorImaging. Band-intensities were quantified using the ImageQuant software (Molecular Dynamics).

## Competing interests

The author(s) declare that they have no competing interests.

## Authors' contributions

CL designed the study, carried out the experiments, and drafted the manuscript. RP participated in design and coordination of the study and helped to draft the manuscript. Both authors read and approved the final manuscript.
